# Giant cell angiofibroma misdiagnosed as a vascular malformation and treated with absolute alcohol for one year: a case report and review of the literature

**DOI:** 10.1186/1477-7819-12-117

**Published:** 2014-04-24

**Authors:** Yue He, Chenping Zhang, Guanglong Liu, Zhuowei Tian, Lizhen Wang, Evagelos Kalfarentzos

**Affiliations:** 1 Department of Oral and Maxillofacial-Head and Neck Oncology, Ninth People’s Hospital, Shanghai Jiao Tong University School of Medicine, 639, Zhi Zao Ju Road, Shanghai 200011, People's Republic of China; 2 Department of Oral Pathology, Ninth People’s Hospital, Shanghai Jiao Tong University School of Medicine, Shanghai, People's Republic of China

**Keywords:** Giant cell angiofibroma, Parotid gland, Parapharyngeal space, Immunohistochemistry

## Abstract

**Purpose:**

To present the clinical, imaging, pathological and immunohistochemical features of giant cell angiofibroma (GCA).

**Case presentation:**

In this paper we report an atypical case of a GCA extending from the parotid to the parapharyngeal space. The lesion was being treated as a vascular malformation for one year prior to surgical removal. We summarize the clinical manifestations, imaging, pathological and molecular features of this rare disease.

After complete surgical removal of the tumor, immunohistochemical analysis revealed strong positivity for the mesenchymal markers vimentin, CD34, CD31 and CD99 in neoplastic cells. Tumor proliferation antigen marker Ki67 was partly positive (<5% of cells). Tumor cells were negative for muscle-specific actin, epithelial membrane antigen, smooth muscle actin, cytokeratin pan, S100, desmin, glial fibrillary acidic protein, myogenin, MyoD1 and F8. The morphological and immunohistochemical profile was consistent with the diagnosis of GCA.

**Conclusion:**

GCA is a rare soft tissue tumor that can easily be misdiagnosed in the clinical preoperative setting. In view of the clinical, pathological and molecular features of the tumor, complete surgical removal is the current optimal treatment option, providing accurate diagnosis and low to minimal recurrence rate.

## Background

Giant cell angiofibroma (GCA) was first described by Dei Tos as a rare soft tissue tumor of the orbit [1]. Since the initial report of the disease in orbital tissue, GCA has been reported in a variety of other extraorbital sites [2-8]. GCA is a benign, mesenchymal lesion showing histological features intermediate between, but distinct from, solitary fibrous tumor (SFT) and giant cell fibroblastoma (GCF) of soft tissue [5].

In this paper, we report a rare case of a patient with a large GCA extending from the right parotid gland to the homolateral parapharyngeal space that was being treated as a vascular malformation for a year prior to surgical removal. The clinical findings, diagnostic procedure followed, treatment and histopathological/immunohistochemical findings are also outlined in this paper.

## Case presentation

### Clinical examination

A 29-year-old male was referred to our outpatient department with a 24-month history of a slowly growing, painless mass of the right parotid gland and submandibular region. He also complained of mild discomfort during swallowing. The patient’s medical history was unremarkable with no previous history of surgery. At the time, he was diagnosed as having a vascular malformation of the right parotid-parapharyngeal region and he had been under treatment for one year with sclerotherapy with no evidence of improvement or stabilization of the tumor.

**Figure 1:**
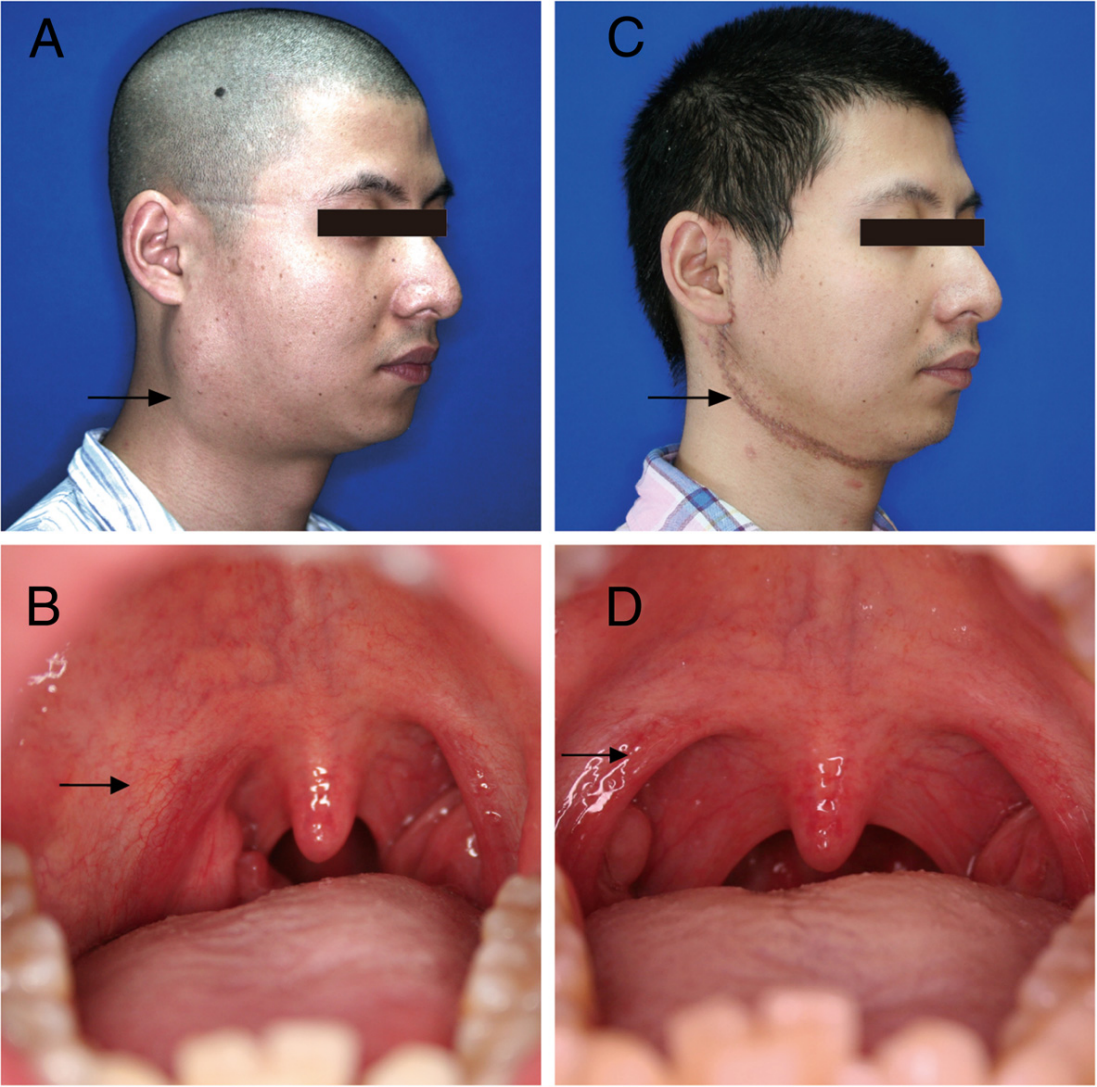
**Clinical view of the tumor. (A)** Preoperative extraoral clinical view of the patient. **(B)** Preoperative intraoral view of the patient. **(C)** Postoperative extraoral clinical view of the patient. **(D)** Postoperative intraoral clinical view of the patient.

Extraoral physical examination at the time of referral was suggestive of a large, mobile, smooth-surfaced, fibroelastic soft tissue mass, which was palpated in the lower pole of the right parotid gland. The lesion extended subcutaneously along the homolateral submandibular and pharyngeal spaces (Figure 1A). On palpation the mass was painless with no evidence of any emanating pulsation. Neurological examination of cranial nerves was normal, and no palpable lymph nodes were present in the neck, supraclavicular or axillary regions.

Upon intraoral examination, there was evidence of the mass bulging to the right oral pharynx with subsequent obliteration of the palatoglossal arch. Deviation of the uvula and soft palate was not evident during opening of the mouth. Covering mucosa of the region was normal in color and texture (Figure 1B). The remainder of the head and neck clinical examination was normal.

### Diagnostic tests

**Figure 2:**
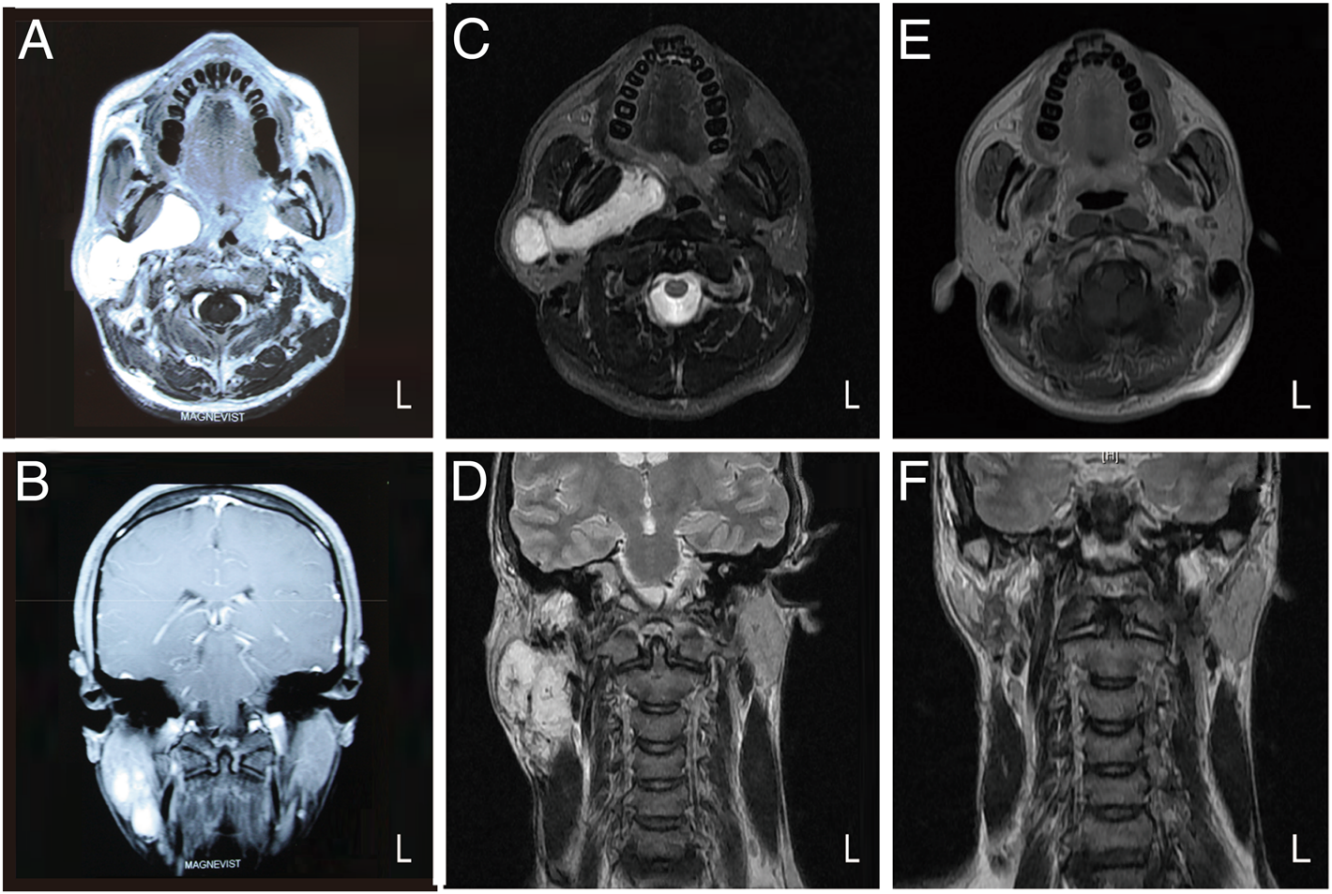
**Giant cell angiofibroma. (A)** Axial T1-weighted MRI of the tumor prior to sclerotherapy. **(B)** Coronal T1-weighted MRI of the tumor prior to sclerotherapy. **(C)** Preoperative axial T2-weighted MRI of the tumor after sclerotherapy. **(D)** Preoperative coronal T1-weighted MRI of the tumor after sclerotherapy. **(E)** Postoperative axial T1-weighted MRI at six months after resection showing no signs of recurrence. **(F)** Postoperative coronal T1-weighted MRI at six months after resection showing no signs of recurrence.

The patient was initially clinically diagnosed with a vascular malformation (VM) of the parotid, submandibular and parapharyngeal spaces based on computed tomography (CT) scan, magnetic resonance imaging (MRI) of the head and neck and a fine needle aspiration biopsy that was indicative of intense hemorrhagic necrosis. At the time, the mass measured approximately 58 × 25 × 47 mm (Figure 2A, B). After unsuccessful treatment with sclerotherapy for one year, a new MRI was performed revealing slow growth of the tumor. MRI was suggestive of an inhomogeneous mass which measured 81 × 34 × 67 mm and extended from the right parotid gland into the right parapharyngeal space. (Figure 2C, D). The signal of the mass was isointense to the circumambient soft tissue in T1W1. Hyperintense signal was demonstrated in T2W1 and T2W1 (Fat suppression) images. There was no sign of infiltration to adjacent soft tissue and no bone erosion was identified on imaging studies. MRI findings were suggestive of a benign parotid gland tumor with involvement of the homolateral submandibular and parapharyngeal spaces.

### Treatment

As mentioned above, clinical history and physical examination were suggestive initially of the diagnosis of VM. Thus, the patient was first treated with a regimen of percutaneous sclerotherapy using ethanol and lauromacrogol for a period of one year without any complications. During this period, no response to sclerotherapy was evident; furthermore the tumor slowly increased in size with no other significant changes in regard to its clinical and radiological consistency.

Since sclerotherapy treatment proved unsuccessful, after discussion and written consent from the patient the decision to surgically remove the tumor was made. The patient was prepared for general anesthesia via nasotracheal intubation. Access to the tumor was feasible through a preauricular incision with a submandibular extension. A skin flap above the parotid fascia and incorporating the platysma muscle was raised with subsequent identification and preservation of the cervicofacial branch of the facial nerve. The tumor appeared to have no obvious infiltration to the surrounding tissues and was excised completely. There was no major intraoperative bleeding. The residual surgical defect was not reconstructed and the wound was closed in layers under suction drainage.

Macroscopically, the tumor was shaped like a dumbbell and contained a single cystic space filled with a yellow-brown sticky fluid.

The patient’s postoperative healing was uneventful. He is on a regular follow up program and six months postoperatively there is no sign of recurrence (Figure 1C, D, Figure 2E, F).

### Histopathology

**Figure 3:**
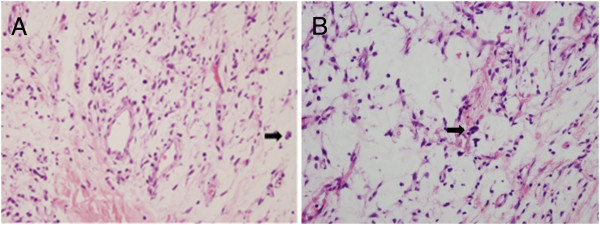
**Histopathological examination. (A)** and **(B)** Microscopic examination revealed a circumscribed, richly vascularized, patternless spindle cell proliferation containing pseudovascular spaces and floret-like multinucleated giant cells (arrow), sometimes lining the pseudovascular spaces, in a variably collagenous or myxoid stroma. (H&Ex400).

Light microscopy examination of the lesion showed increased cellularity, with spindled and elongated cells intermingled with variable amounts of capillary-sized blood vessels, pseudovascular spaces, and floret-like multinucleated giant cells (Figure 3). At high magnification, the spindle-shaped tumor cells had plump nuclei and pale eosinophilic cytoplasm. No mitoses were identified.

**Figure 4:**
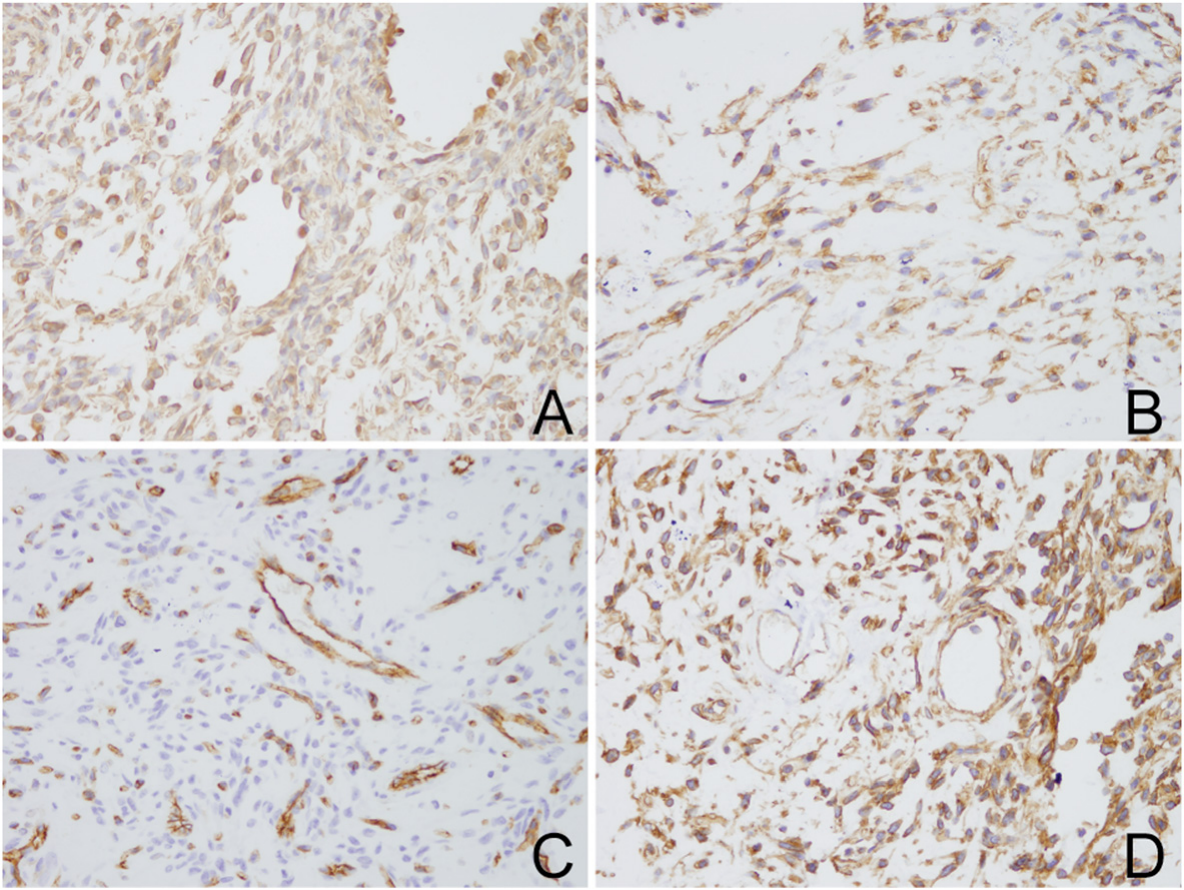
**Immunohistochemical analysis revealed strong positivity for the mesenchymal markers. (A)** Vimentin. **(B)** CD34. **(C)** CD31. **(D)** CD99.

Immunohistochemistry was performed and the mesenchymal markers vimentin, CD31, CD34, CD99 were positive in spindle cells and giant cells. Tumor proliferation antigen marker Ki67 was partly positive (<5% of cells). Muscle-specific actin (MSA), epithelial membrane antigen (EMA), smooth muscle actin (SMA), cytokeratin pan (CKP), S100, desmin, CD31, glial fibrillary acidic protein (GFAP), myogenin, MyoD1 and F8 were negative (Figure 4). The morphology and immunohistochemical profile were consistent with the diagnosis of GCA.

## Discussion

GCA is a rare tumor of soft tissues. Although it was firstly described as a distinct orbital neoplasm by Dei Tos [1], until now there are approximately 26 various extraorbital site cases reported in the literature [2-18] (Table 1). By taking into account the orbital GCA cases and extraorbital head and neck cases we could assume that GCA may have a predilection for the head and neck region.

**Table 1:** Known reported giant cell angiofibroma extraorbital cases

**Head and neck area**		**Other anatomic areas**	
Subcutaneous neck area	1	Cutaneous (thigh)	1
Occipital region	2	Hip	1
Retroauricular area	1	Forearm	1
Parotid	1	Vulva	1
Submandibular region	1	Retroperitoneum	1
Parapharyngeal space	1	Back	3
Oral buccal mucosa	3	Mediastinum	1
Tongue	1	Axilla	1
Vocal cord	2	Groin/Inguinal region	3

Source: References [2-18].

The tumor may grow rapidly, simulating malignancy, or may have an indolent course with slow growth over many years. GCA most often occurs in middle-aged adults (median age 45 years). In regard to sex predilection of this tumor, some reports suggest that orbital GCA is more common in men, while extraorbital locations are more common in women [6].

Gonzalez-Perez *et al*. were the first to report a GCA of the parapharyngeal region in a 25-year-old female patient [6]. Both GCA cases presented with similar clinical characteristics of a benign non-infiltrative slow growing tumor. In both cases a differential diagnosis of parotid gland, fibrous or vascular tumor was made. In our case, the more cystic (pseudocystic) nature of the tumor in addition to the lack of other evidence from the fine needle aspiration biopsy, led to the misdiagnosis of the tumor as a VM. Despite the positive results for CD31 in our case, histological and immunohistochemical findings were also similar in both cases.

The pathogenesis of the tumor remains unclear. There are currently two papers in the literature trying to associate an orbital case of GCA with mutations on chromosome 6q13 [18] and another extraorbital case with an associated translocation t(12;17) [14].

In the clinical setting during preoperative management, diagnosis of GCA may be virtually impossible. Besides the fact that GCA is an extremely rare tumor it also shares many clinical and radiological features with other soft tissue tumors of the head and neck. Furthermore the pseudovascular spaces that are present in large GCAs may associate it with tumors of vascular origin, as in our case. The multitude of soft tissue tumors of the region that need to be differentially diagnosed from GCA, can be roughly depicted in an abbreviated summary of the World Health Organization (WHO) system for classifying soft tissue tumors on the basis of tissue type and biologic potential (Table 2) [19].

**Table 2:** Summary of World Health Organization (WHO) classification of soft tissue tumors of the neck

**Histologic type**	**Benign**	**Intermediate, locally aggressive**	**Intermediate, rarely metastasizing**	**Malignant**
Adipocytic	Lipoma and its variants (lipoblastoma, hibernoma, lipomatosis)	Atypical lipomatous tumor, well-differentiated liposarcoma	…	Liposarcoma
Fibroblastic/myofibroblastic	Fibromatosis colli, myofibroma, giant cell angiofibroma	Desmoid-type fibromatosis	Solitary fibrous tumor hemangio-pericytoma, inflammatory myofibroblastic tumor (inflammatory pseudotumor)	Fibrosarcoma
So-called fibrohistiocytic	Benign fibrous histiocytoma, diffuse-type giant cell tumor (pigmented villonodular synovitis)	…	Giant cell tumor of soft tissues	Malignant fibrous histiocytoma (undifferentiated pleomorphic sarcoma)
Skeletal muscle	Rhabdomyoma	…	…	Rhabdomyosarcoma
Smooth muscle	Leiomyoma, angioleiomyoma	…	…	Leiomyosarcoma
Vascular	Hemangioma, lymphangioma	Kaposiform hemangioendothelioma	Kaposi sarcoma	Angiosarcoma
Perivascular	Glomus tumor, myopericytoma	…	…	Malignant glomus tumor
Chondro-osseous	Soft tissue chondroma	…	…	Mesenchymal chondrosarcoma, extraskeletal osteosarcoma
Uncertain differentiation	Myxoma	…	Ossifying fibro-myxoid tumor	Synovial sarcoma, alveolar soft part sarcoma, primitive neuroectodermal tumor, Ewing sarcoma

Source- Reference [19].

Vascular malformation is a collective term for many different disorders of the vasculature. It can be a disorder of capillaries, arteries, veins and lymphatics or a combination of those. A VM consists of a clew of deformed vessels, due to an error in vascular development. However, endothelial turnover is stable in these defects. Congenital VMs are always present at birth, although they are not always visible. In contrast to vascular tumors, vascular malformations do not have a growth phase, nor an involution phase, they tend to grow proportionately with the child, never regressing and persisting throughout life [20]. Vascular malformations can be divided in slow-flow VM, fast-flow VM and complex-combined VM. Clinically, diagnosis of superficial VM is not difficult, deep VM should be determined by clinical examination with identification of postural movement and a positive puncture test. Ultrasound and MRI can aid significantly in the diagnosis of VM, while angiography is usually reserved for treatment planning.

In our case, the patient had a slow growing tumor in the head and neck region over a period of more than two years. MRI findings were suggestive of a benign non-infiltrating tumor with heterogeneous signal intensity extending from the parotid to the parapharyngeal space which demonstrated intense enhancement after the administration of contrast material. Overall, the cystic nature of the tumor in conjunction with excess blood material in the fine needle aspirate led to the misdiagnosis of this GCA case as a vascular malformation. Sclerotherapy, as expected, had no effect on the lesion which in fact continued to slowly grow. Final diagnosis was only feasible after referral of the patient for surgical removal and subsequent histopathological and immunohistochemical testing of the tumor.

GCA is typically regarded as a non-invasive neoplasm with no potential for metastatic disease compared to solitary fibrous tumor which may rarely metastasize [19]. There is controversy regarding the potential of the tumor for recurrence. Since there have not been enough reports of GCA to allow us to come to any definitive conclusions regarding its long-term behavior, complete surgical removal and long-term follow up was considered the optimal treatment option.

Diagnosis of GCA is usually made after resection and immunohistochemical analysis. Histological characteristics of GCA include: (1) the presence of homogenous irregularly organized proliferating cells that are round or oval shaped (2) the stromal region that is often collagenized or myxoid and (3) the tumor is richly vascularized and contains pseudovascular spaces in the presence of interstitial hemorrhage. The pseudovascular spaces are lined with multinucleated giant cells [11].

The immunohistochemical features of GCA include positive staining for CD34, CD99, vimentin, variable bcl2 and negative staining for CD31, CD68, c-kit/CD117, muscle specific actin, S100, desmin [7,21].

In our case, positive staining for CD34 in combination with the presence of giant cells and pseudovascular spaces confirmed the diagnosis of GCA. A controversial finding was the unexpected positivity of CD31 in the majority of cells compared to previous reports [7,21]. Clinically, this immunohistochemical finding may be interpreted by the more vascular nature of the tumor and it may provide a tool for differential diagnosis of GCA from SFT in the future. Nevertheless several other soft tissue tumors may present with similar findings. For example, GCA might be closely related to GCF, multinucleate cell angiohistiocytoma (MCA), SFT and VM.

GCF is usually differentially diagnosed from GCA by the fact that it is usually a poorly circumscribed lesion invading the dermis and subcutis of somatic soft tissues. The tumor, which typically occurs within the first decades of life, is rarely described in middle-aged and older patients. It most commonly affects the trunk [22]. Although GCF is composed of CD34 positive spindle and stellate shaped cells including multinucleated floret-like giant cells and tumor cell-lined pseudovascular spaces, it has infiltrative margins and has less conspicuous cellularity and vasculature. Clinically, GCF appears to be a more aggressive lesion than GCA in that up to 50% of cases locally recur [3].

MCA is a rare benign proliferation of the skin of unknown etiology. Clinically, MCA shows a predilection for adult women (F:M ratio 3:1) and manifests as a single or multiple firm, red-brown or violaceous papules with a smooth, or occasionally, scaly surface [23]. Histological examination shows a vascular proliferation predominantly of capillaries and veins in the upper and mid dermis with lymphohistiocyte infiltration. Multinucleate giant cells are arranged in a ring-like or overlapping pattern, with a positive staining for factor XIIIa [24].

The CD34-positive cell neoplasm most closely aligned to GCA is the SFT, which was first described in the pleura but has since been reported in many locations. In approximately half of all described cases it is located in the subcutaneous tissue, but also in deep soft tissue of the extremities, or in the head and neck region, the thoracic wall, mediastinum and abdominal cavity. SFTs on CT scans show moderate to intense enhancement in a well circumscribed mass. Histologically, SFTs show a patternless spindle cell proliferation in a collagenous stroma, although myxoid areas are not uncommon, and a hemangiopericytomatous vascular pattern that is not seen in GCA. Giant cells and pseudovascular spaces are infrequent and serve to distinguish this tumor type from GCA. Both are immunoreactive for CD34 and CD99 [16,25,26].

There is a lot of debate in the literature concerning the classification of GCA. Although many authors believe GCA to be a distinct tumor with distinct histological and immunohistochemical characteristics, others support the opinion that this may be a variant of either SFT or GCA. In fact, the idea that GCA may be a variant of SFT is further supported by WHO’s latest reclassification (2013) [27], where GCA is considered a synonym for extrapleural SFT rather than being a separate entity. In this reclassification GCA, SFT, hemangiopericytoma and lipomatous hemangiopericytoma are all interconnected and are included under the same category of ‘extrapleural SFT’.

## Conclusion

In conclusion, GCA is a rare benign tumor which mainly affects soft tissue(s) in the head and neck area. Clinical or radiological diagnosis alone is very difficult since clinical characteristics and radiological features of the tumor resemble those of many other benign soft tissue tumors. In particular, for cases resembling vascular malformations as in our report, clinicians should be aware of this entity to avoid unnecessary delay and treatment. Surgery is the main treatment. Microscopic examination and immunohistochemical analysis provide the only means of diagnosis mainly by identification of giant cells and pseudovascular spaces in conjunction with positivity for vimentin and CD34. Classification of GCA is still under question in our opinion. Further reports and studies of this tumor are needed to define the pathophysiology and understanding of the tumor.

## Consent

Written informed consent was obtained from the patient for publication of this case report and any accompanying images. A copy of the written consent is available for review by the Editor-in-Chief of this journal.

## Abbreviations

CKP: cytokeratin pan; CT: computed tomography; EMA: epithelial membrane antigen; GCA: giant cell angiofibroma; GCF: giant cell fibroblastoma; GFAP: glial fibrillary acidic protein; H&E: hematoxylin and eosin; MCA: multinucleate cell angiohistiocytoma; MRI: magnetic resonance imaging; MSA: muscle-specific actin; SFT: solitary fibrous tumor; SMA: smooth muscle actin; VM: vascular malformation; WHO: World Health Organization.

## Competing interests

The authors declare that they have no competing interests.

## Authors’ contributions

YH and EK wrote the paper. LW carried out the histological and immunohistochemical studies of the surgical specimens. GL and ZT were involved in collecting the clinical, imaging and laboratory data of the patient. CZ edited and modified the paper. All authors read and approved the final manuscript.
